# FGF23 Producing Mesenchymal Tumor

**DOI:** 10.1155/2014/492789

**Published:** 2014-02-03

**Authors:** Lucyna Papierska, Jarosław B. Ćwikła, Waldemar Misiorowski, Michał Rabijewski, Krzysztof Sikora, Hubert Wanyura

**Affiliations:** ^1^Clinic of Endocrinology, Medical Center of Postgraduate Education, Warsaw, Poland; ^2^Department of Radiology, The Faculty of Medical Sciences, University of Warmia and Mazury, Olsztyn, Poland; ^3^Department of Internal Diseases, Diabetology and Endocrinology, Medical University of Warsaw, Warsaw, Poland; ^4^Department of Clinical Pathology, Central Clinical Hospital Ministry of Internal Affairs and Administration, Warsaw, Poland; ^5^Clinic of Cranio-Maxillofacial Surgery, Medical University of Warsaw, Warsaw, Poland

## Abstract

A 40-year-old patient was referred to Clinic of Endocrinology due to hypophosphatemia causing pain, cramps, and weakness of muscles. Moreover, his bone mineral density was very low. The previous treatment with phosphorus and active vitamin D metabolites was ineffective. In lab tests the hypophosphatemia, hyperphosphaturia, and elevated FGF23 levels were found. Somatostatin receptor scintigraphy (SRS) showed increased radiotracer uptake in the right maxillary sinus and CT scans confirmed presence of tumor in this localization. Biopsy and cytological examination created suspicion of mesenchymal tumor—glomangiopericytoma. Waiting for surgery the patient was treated with long acting Somatostatine analogue, and directly before operation short acting Octreotide and intravenous phosphorus were used. Histology confirmed the cytological diagnosis and the phosphatemia return to normal values in 10 days after the tumor removal.

## 1. Case Report

A 40-year-old man was referred to Clinic of Endocrinology due to prolonged, deep hypophosphatemia causing pain, cramps, and weakness of proximal muscles. One year before, during the previous hospitalization (in neurological ward) primary muscle disease had been excluded and diagnosis of osteomalacia had been established. Diagnosis was made on basis of symptoms, low calcium and phosphorus level (2.1 mmol/L and 0.6 mmol/L, resp.), and very low 24 h calcium urine excretion (80 mg/24 h). PTH was then surprisingly normal that is, 57 pg/mL (normal ranges 15–65 pg/mL), and 25OHD3 level was undetectable (<4 ng/mL). There were no fractures in patient's medical history; however bone mineral density was very low (in all localizations *T*-scores and *Z*-scores <−3). Patient was treated for more than one year with calcitriol 1 *μ*g, alfacalcidol 1 *μ*g, calcium 1000 mg, and phosphorus 1500 mg per day. This medication had no effect on serum concentrations of phosphorus and only a moderate effect on clinical symptoms. Patient was under dental treatment (caries and periodontitis). At admission the levels of CPK, calcium, and PTH were normal; 25OHD3 level raised to 29.1 ng/dL (normal ranges 30–80 ng/dL) despite using only little amount of D3 contained in Ca/D3 preparations apart from active metabolites. Alkaline phosphatase (AP) was slightly elevated (137 U/L; normal ranges 40–129 U/L) and serum phosphorus concentration was very low (0.41 mmol/L; normal ranges 0.81–1.45 mmol/L). 24 h urine collection showed high phosphorus excretion (66.5 mmol/24 h, normal ranges 12.00–65.00 mmol/24 h). Bone mineral density was still very low: lumbar spine *T*-score = −3.1 and femoral neck *T*-score = −3.9. The level of phosphaturic agent FGF23, assessed by Enzyme-linked immunosorbent assay (ELISA) was high (120 pg/mL; normal ranges 8.2–54.3 pg/mL). Therefore tumor-induced hypophosphatemia was diagnosed and searching of FGF-producing tumor was initiated.

**Figure 1:**
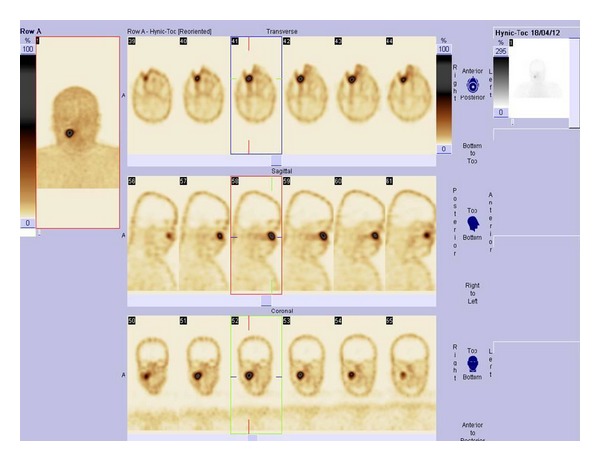
SRS: increased radiotracer uptake in the area of right maxillary sinus.

**Figure 2:**
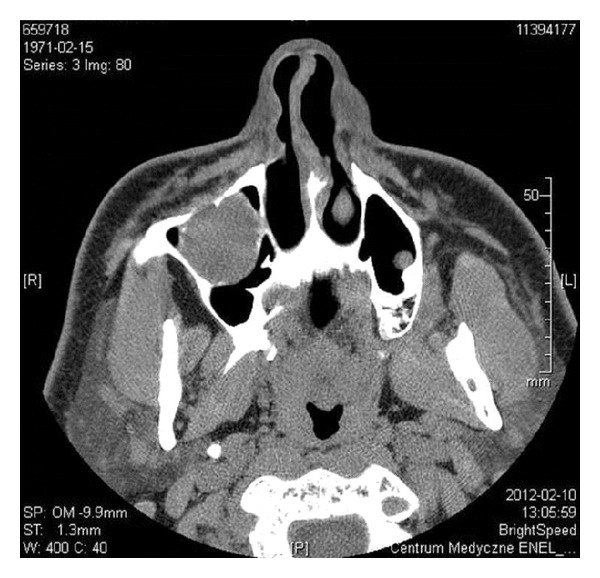
CT: 3 cm diameter, oval hypodense tumor in right maxillary sinus.

**Figure 3:**
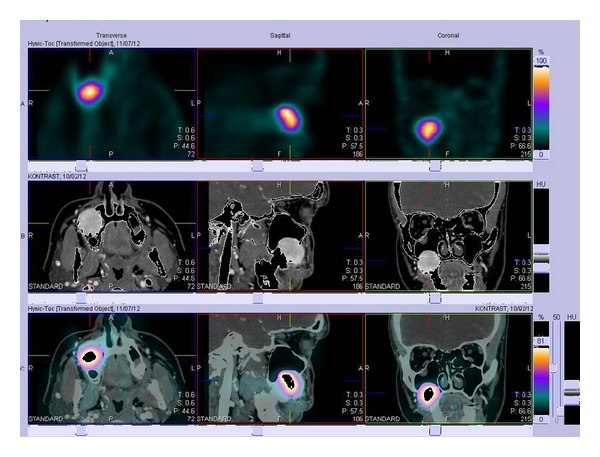
Fusion of CT and SRS scans: accumulation of radiotracer in the tumor.

**Figure 4:**
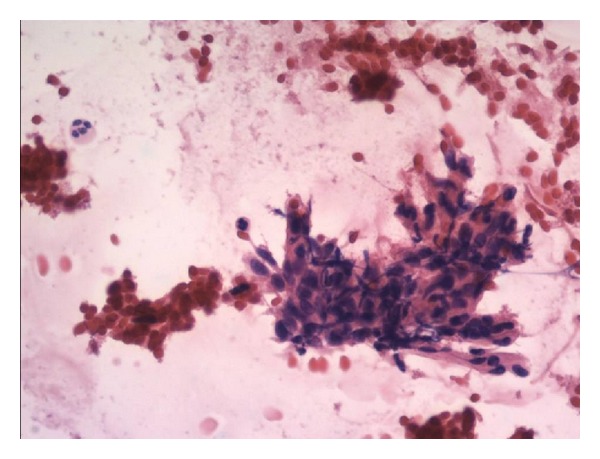
Cytologic smear: groups of monomorphic elongated cells, with eosinophilic cytoplasm and hyperchromatic spindly shaped nuclei with mild atypia.

The CT scans of chest and abdomen did not reveal any changes, also NMR images of abdomen were normal. Somatostatin receptor scintigraphy (SRS), performed using ^99m^Tc-[HYNIC,Tyr3]-octreotide, 700 MBq (IAE: Institute of Atomic Energy: Polatom, PL), showed increased radiotracer uptake in the right maxillary sinus (Figure 1). On CT the 3 cm diameter, oval hypodense tumor was found in this localization (Figure 2), and fusion of CT and SRS scans confirmed the localization of the labeled Octreotide uptake in it (Figure 3). Biopsy of lesion showed groups of monomorphic elongated cells, with eosinophilic cytoplasm and hyperchromatic spindly shaped nuclei with mild atypia (Figure 4). On the basis of these findings the suspicion of glomangiopericytoma was determined. Patient was referred to Clinic of Cranio-Maxillofacial Surgery.

On the basis of confirmed Octreotide uptake in tumor, we ordered long acting Somatostatin analogue (Sandostatin LAR 30 mg). Two days after the injection phosphorus level increased from 0.5 to 0.68 mmol/L. Directly before the surgery, we additionally used intravenous phosphorus and repeated injection of Somatostatin (Sandostatin) twice daily—phosphorus level increased to 0.75 mmol/L. After the removal of tumor serum phosphorus levels increased to normal values in 10 days on oral preparations only. Histology confirmed the diagnosis of glomangiopericytoma. Unfortunately, we have not had an opportunity to repeat the FGF23 measurement after operation as in Poland these assays are made very rarely in highly specialized medical centers.

Six months after the operation the phosphorus level achieves 1.1 mmol/L. The bone mineral density raised significantly: DEXA measurements revealed normal BMD in lumbar spine (*T*-score = 0.4) and osteopenia (*T*-score = −1.7) in femoral neck. Muscle function is normal, all symptoms reported previously retreated. Repeated SRS-scans did not show any lesions with high radiotracer uptake. The only medications at present are vitamin D3 2000 IU and calcium preparations (500 mg of elemental calcium) per day.

## 2. Discussion

**Figure 5:**
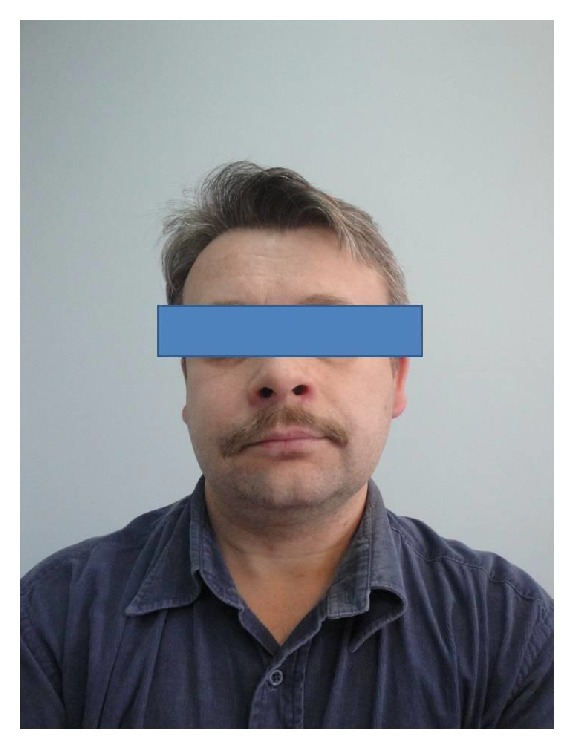
Obvious facial asymmetry caused by tumor in the right maxillary sinus.

Tumor-induced osteomalacia (TIO) is a rare condition associated with hypophosphatemia, myopathy, and systemic bone demineralization caused by renal phosphate wasting in conditions of excessive production of fibroblast growth factor 23 (FGF23) by neoplasmatic, most often, benign lesions. FGF23 producing tumors are usually very small; therefore their identification can be difficult. The second reason for difficulties of tumor finding is wide variety of its localization—all regions and organs of body could be the site of it [1]. Authors describing such cases emphasize a role of very careful physical examination of patients as mesenchymal tumor can be localized in subcutaneous tissue and therefore available for palpation [2]. These tumors were also reported in the jaws and sinonasal area [3–5]. In general, however, this localization was regarded as rather rare: in 2004, in retrospective analysis of 109 cases, Folpe found only 13 FGF-producing mesenchymal tumors localized in the head and neck region [6]. In recently described series of 39 patients with TIO Jiang and coworkers found majority (56%) of lesions in lower extremities but tumors localized in the head constituted 31% of all cases [7]. Due to variety of FGF-producing tumors localizations, CT and NMR should be preceded by functional imaging, that is, SRS or PET [8, 9]. However if our patient was accurately examined, physician should notice facial asymmetry (Figure 5), which was absent on former photo in his identity card. This change was explained by patient and his family as a consequence of current dental treatment.

After the proper recognition of tumor site of our patient a problem has occurred: according to anesthesiologist's wish we had to achieve at least near to lower range of limits of phosphorus blood concentration. It was necessary for safety of anesthesia and proper function of respiratory muscles after the extubation. Basing on high Octreotide uptake in tumor, we decided to use the long acting analogue of Somatostatine, followed by short acting Octreotide in order to amplify the effect of medication on the phosphorus blood levels. We found similar cases in the literature; unfortunately most authors described that Sandostatin analogues allowed to decrease FGF-levels but failed to restore normal phosphorus level [10–12]. In our patient phosphorus level after the Octreotide injection was also still low; however it increased from extremely low values to values near normal ranges with evident improvement of clinical outcomes. The similar effect in patient with mesenchymal tumor in left thigh was described by Seufert et al. [13]. The improvement of phosphorus level increased safety of general anesthesia during surgery. In our opinion the use of Octreotide is therefore justified and proved to be helpful in preoperative treatment and then could be recommended in cases like ours, before the tumor removal.
